# Fish Suppressors of Cytokine Signaling (SOCS): Gene Discovery, Modulation of Expression and Function

**DOI:** 10.1155/2011/905813

**Published:** 2011-12-13

**Authors:** Tiehui Wang, Bartolomeo Gorgoglione, Tanja Maehr, Jason W. Holland, Jose L. González Vecino, Simon Wadsworth, Christopher J. Secombes

**Affiliations:** ^1^Scottish Fish Immunology Research Centre, Institute of Biological and Environmental Sciences, University of Aberdeen, Aberdeen AB24 2TZ, UK; ^2^EWOS Innovation AS, 4335 Dirdal, Norway

## Abstract

The intracellular suppressors of cytokine signaling (SOCS) family members, including CISH and SOCS1 to 7 in mammals, are important regulators of cytokine signaling pathways. So far, the orthologues of all the eight mammalian SOCS members have been identified in fish, with several of them having multiple copies. Whilst fish CISH, SOCS3, and SOCS5 paralogues are possibly the result of the fish-specific whole genome duplication event, gene duplication or lineage-specific genome duplication may also contribute to some paralogues, as with the three trout SOCS2s and three zebrafish SOCS5s. Fish SOCS genes are broadly expressed and also show species-specific expression patterns. They can be upregulated by cytokines, such as IFN-**γ**, TNF-**α**, IL-1**β**, IL-6, and IL-21, by immune stimulants such as LPS, poly I:C, and PMA, as well as by viral, bacterial, and parasitic infections in member- and species-dependent manners. Initial functional studies demonstrate conserved mechanisms of fish SOCS action via JAK/STAT pathways.

## 1. Introduction

Cytokines have essential roles in the development, differentiation, and function of the immune response. Following receptor ligation cytokines, including interleukins (ILs), interferons (IFNs), and haematopoietic growth factors, activate the Janus kinase-signal transducer and activator of transcription (JAK-STAT) pathways to elicit downstream effects in responsive cells. The intracellular suppressors of cytokine signaling (SOCS) family members are emerging as one of the most important regulators of these pathways [1]. So far eight members of the SOCS family, including cytokine-inducible SRC homology 2- (SH2-) domain-containing protein (CISH) and SOCS1–7, have been identified in mammals, all of which are structurally characterized by a central SH2 domain and a conserved C-terminal motif named as the SOCS box [2]. Most SOCS proteins are induced by cytokines and therefore act in a classical negative-feedback loop to inhibit cytokine signal transduction. However, they are also induced by various other stimuli, such as pathogen-associated molecular patterns (PAMPs), and bacterial, viral, and parasitic infection [3].

Many important cytokines have been identified in teleost fish in the last decade aided by the sequenced genomes of a few model teleosts, that is, tetraodon (*Tetraodon nigroviridis*), zebrafish (*Danio rerio*), fugu (*Takifugu rubripes*), stickleback (*Gasterosteus aculeatus*), and medaka (*Oryzias latipes*), and large expressed sequence tag (EST) projects in a few economically important species such as the salmonids Atlantic salmon (*Salmo salar*) and rainbow trout (*Oncorhynchus mykiss*) [4]. The cytokines discovered in teleosts include proinflammatory cytokines such as IL-1*β*, IL-6, IL-8, IL-11, and tumour necrosis factor-*α* (TNF-*α*) and anti-inflammatory cytokines such as IL-10, transforming growth factor-*β*1 (TGF-*β*1), and a novel IL-1 family member discovered in fish that antagonises the activity of IL-1*β* [4]. Important initiators and effectors of adaptive immune responses have also been cloned, such as IL-2, IL-4/13-like, IL-7, IL-12, IL-15, IL-17 family members, IL-18, IL-20, IL-21, IL-22, type-I IFNs, and IFN-*γ* [5]. The recombinant proteins have been produced for a few of these cytokines, for example, IL-1*β* [6], IL-2 [7], IL-6 [8], IL-8 [9], IL-10 [10], IL-4/13-like, IL-15 [11], IL-21 [12], IL-22 [13], TNF-*α* [14, 15], type-I IFNs [16], and IFN-*γ* [17, 18], to begin to validate their functional relevance. One intriguing finding is that many homologues of mammalian cytokines and their receptors have duplicates (i.e., more than one isoform) in fish [5].

Most recently the fish immunology research community has begun to elucidate the negative regulators of cytokine signalling. All the mammalian SOCS family members have been identified in fish, with some additional novel teleost members that have likely resulted from fish-specific gene/genome duplication events [19]. In this paper, we will first describe the SOCS family members that have been discovered in fish and present new data on the cloning of additional members, that is, CISHb and two further SOCS2 sequences, in rainbow trout. The relationship between the proposed fish SOCS8 and CISH, and fish SOCS9 and SOCS5 will then be discussed. Next results on the expression of fish SOCS genes will be presented, with a focus on new data on the modulation of SOCS gene expression by viral, bacterial, and parasitic infections in salmonids. Finally, the possible function of fish SOCS proteins in the context of duplicated cytokines and SOCS molecules in fish will be discussed.

## 2. Fish SOCS Gene Discovery

### 2.1. Fish CISH and SOCS8

**Figure 1:**
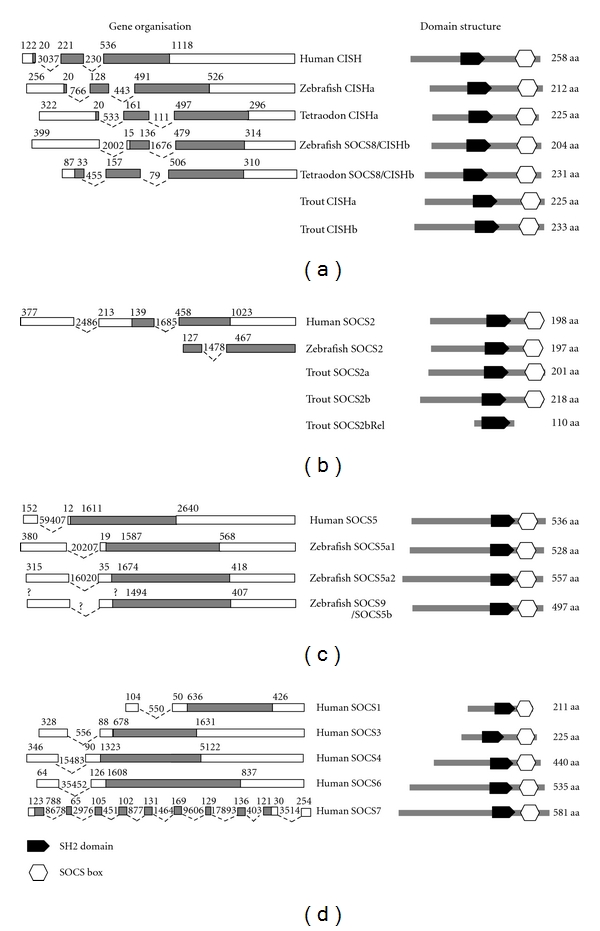
Gene organisation and domain structure of CISH (a), SOCS2 (b), SOCS5 (c), and other human SOCS molecules (d). The gene organisation is extracted from the publication by Jin et al. [20] except the tetraodon CISH gene that was derived from the cDNA sequence (acc. no. EF195758) and its genome sequence (at the website http://www.genoscope.cns.fr/blat-server/cgi-bin/tetraodon/). Open boxes represent the untranslated regions, whilst grey boxes represent the coding regions, with sizes given above. Dotted lines show the intron positions and sizes. The domain structure was predicted using the SMART program (at the website http://smart.embl-heidelberg.de/).

CISH genes have been described in the five model fish (zebrafish, tetraodon, fugu, medaka, and stickleback) for which a genome sequence is available [20], as well as in rainbow trout [21]. CISH sequences have also been submitted to the database from Atlantic salmon (Salmon, acc. nos. B9EPA9 and B5XCB4), rainbow smelt (*Osmerus mordax*) (Smelt, acc. no. C1BIN3), and grass carp (*Ctenopharyngodon idella*) (gCarp, acc. no. D3Y197). Another sequence has also been described in the five model fish that has high homology to CISH and was designated as fish-specific SOCS8 based on its predicted unique gene organisation. The CISH genes in tetrapods and fish have a three- (coding) exon/two-intron gene organization, whilst the fish SOCS8 was believed to be encoded by two exons, with an intron in the 5′-untranslated region (UTR) [20]. Both fish CISH and SOCS8 molecules as well as tetrapod CISH have similar domain structures including the central SH2 domain and the C-terminal SOCS box (Figure 1(a)).

No SOCS8 sequence has yet been reported in other fish species. We have analyzed for potential SOCS8 genes in other fish species using the BLAST suite of programs in the databases at NCBI (http://blast.ncbi.nlm.nih.gov/Blast.cgi) [22]. Two expressed sequence tags (EST) (accession numbers: EV382534, EV382535) from the same clone from a mixed tissue library of sockeye salmon *Oncorhynchus nerka* (Sockeye) were identified that contained an open reading frame (ORF). The translation from this clone showed 41.8–47.1% identity to tetrapod CISH and similar identities (49.8–56.9%) to both fish CISH and SOCS8 (Table 1). As the sockeye salmon sequence showed only 56.3% identity to its close relative trout CISH, it may be transcribed from a salmonid SOCS8 gene.

To isolate the trout SOCS8 gene, primers were designed at the 5′-UTR of the sockeye salmon sequence and used for 3′-RACE using trout liver SMART cDNA as described previously [23, 24]. A ~1.2 kb band (as revealed on an agarose gel) was amplified, cloned, and sequenced, which contained a partial 5′-UTR, an ORF for 233 amino acids (aa), and the 3′-UTR with a poly A signal 13 bp upstream of the poly A tail (acc. no. FR873795). The trout translation showed 98.7% identity to the sockeye salmon sequence as calculated using the MatGAT program [25]. Both the trout and sockeye translations showed similar identities (49.5–55.3%, Table 1) to CISH and SOCS8 molecules from the model fish but highest identities (55.0–57.3%) to CISH from salmonids. The tetrapod CISHs share comparable identities to both fish CISH (36.8–44.4%) and fish SOCS8 (37.5–47.1%, Table 1). Thus, the homology analysis (shown in Table 1) does not sufficiently separate the fish CISH and SOCS8 into two categories but rather they appear to be fish paralogues of tetrapod CISH. From new evidence on the SOCS8 gene organisation, synteny, and phylogenetic tree analysis that will be described below, we suggest to rename the so called fish SOCS8 as CISHb and the published fish CISH as CISHa.

**Figure 2:**
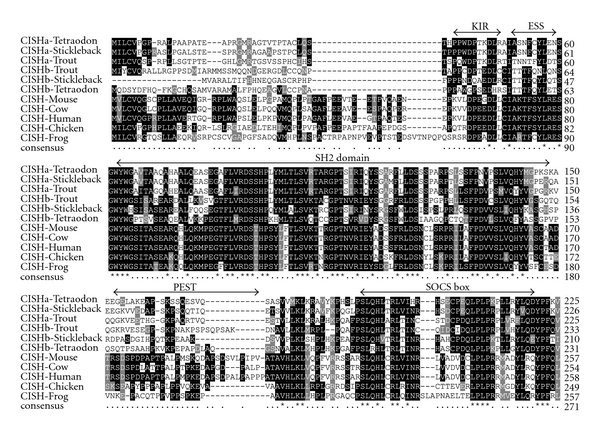
Alignment of CISH molecules from selected tetrapod and fish species. The multiple alignment was produced using the ClustalW2 program and box shaded. Dashes (–) indicate gaps in the alignment. The location of the KIR, ESS region, SH2 domain, and SOCS box are indicated above the alignment. Putative PEST sequences between the SH2 domain and the SOCS box predicted by the epestfind program (http://emboss.bioinformatics.nl/cgi-bin/emboss/epestfind) and present in mammalian and amphibian CISH were missing in fish and bird CISH.

The SH2 domain and SOCS box are critical for SOCS function. The SH2 domain in CISH and SOCS2 binds to a cytokine receptor cytoplasmic domain to compete with STAT-SH2 domains for specific receptor phosphotyrosine residues. The SOCS box motif, by binding to an E3 ubiquitin-ligase complex, ubiquitinates the associated proteins targeting them for proteasomal degradation [26]. In addition, the kinase inhibitory region (KIR) located at the N-terminal and adjacent to the SH2 domain in SOCS1 and 3 is required for inhibition of JAK kinase activity [27], and the extended SH2 domain (ESS) is critical for phosphotyrosine binding [28]. The ESS forms a 15-residue alpha helix, which directly contacts the phosphotyrosine-binding loop and determines its orientation. An alignment of selected molecules from tetrapod CISH and fish CISHa and CISHb was produced using ClustalW2 software [29]. A good conservation was seen at the KIR, ESS region, the SH2 domain, and the SOCS box among all the molecules from tetrapods and fish (Figure 2). It is noticeable that the N-terminal is quite divergent between CISH molecules from mammals, birds, amphibians, and fish, as is the PEST domain. PEST sequences are rich in proline (P), glutamate (E), serine (S), and threonine (T) and are thought to signal for rapid proteolytic degradation [30]. Several mammalian SOCS proteins contain putative PEST sequences [31] suggesting a common mechanism for regulation of SOCS protein levels. Putative PEST sequences can be predicted using the epestfind program (http://emboss.bioinformatics.nl/cgi-bin/emboss/epestfind) in mammalian CISH, as well as in frog CISH between the SH2 domain and the SOCS box, but are missing in CISH molecules from fish and birds (Figure 2).

The trout and sockeye salmon CISHbs are longer (233 aa) compared to the previously predicted model fish CISHbs (SOCS8), for example, 204 aa for zebrafish CISHb, 210 aa for stickleback and medaka CISHbs, and 214 aa for tetraodon and fugu CISHbs. In addition, only two coding exons have been identified in the five model fish, although a third exon is present containing most of the 5′-UTR sequence, rather than three coding exons as seen in CISH [20]. Our BLAST search analysis has identified three full-length tetraodon cDNA sequences (acc. nos. EF195758, CR704107 and CR693174) that encode an identical SOCS8/CISHb protein except that all the three sequences have an in-frame upstream ATG from the predicted start codon [20]. Two additional tetraodon cDNA sequences (acc. nos. CR727386 and CR727861) have also been found that have translations with 98% and 97% amino acid identities to the reported CISHb that are likely the result of polymorphism or sequence error. More importantly, all the five sequences have an in-frame stop codon nine codons upstream of the first ATG. It is known that ribosomes of eukaryotes scan mRNA from the 5′ end towards the 3′ end and initiate translation usually at the first encountered AUG triplet, although the ribosome sometimes ignores the first AUG and initiates translation at the next found AUG triplet, a phenomenon known as “leaky scanning” [33]. There is no evidence of “leaky scanning” in any SOCS genes; thus, the tetraodon CISHb protein could be translated from the first ATG into a protein of 231 aa, a size comparable to trout and sockeye salmon CISHbs. With this information, when the cDNA sequence is compared to its genomic sequence at the website http://www.genoscope.cns.fr/blat-server/cgi-bin/tetraodon/, it shows that the tetraodon CISHb indeed has a three- (coding) exon/two-intron structure, as seen in tetrapod CISH and the known fish CISHa (Figure 1(a)).

The gene organisation of salmonid CISHb genes will be known with the release of the salmon genome sequence. It is likely that the trout and sockeye salmon CISHb gene will have a similar gene organisation to that seen in tetraodon in light of the aa length. However, we could not confirm this gene organisation in fugu, a close relative of tetraodon, as well as in zebrafish, medaka, and stickleback that may indeed only have two coding exons as predicted by Jin et al. [20]. Thus, the CISHb genes in these species encode an N-terminal shortened CISH paralogue.

**Figure 3:**
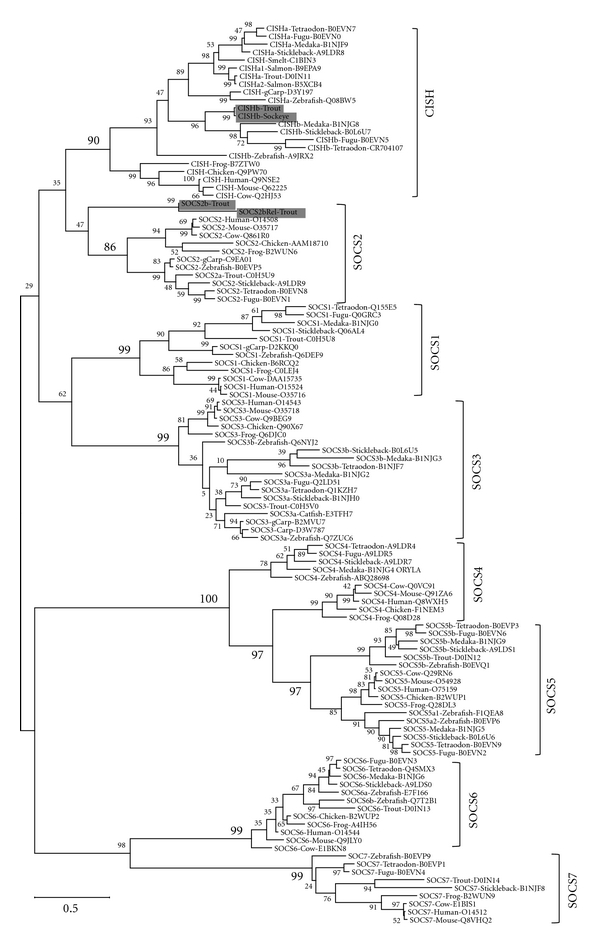
An unrooted maximum likelihood (ML) phylogenetic tree of the SOCS family members from tetrapods and fish. The tree was constructed based on a multiple alignment of all the known fish SOCS family members and selected molecules from mammals, amphibians, and birds, using the Jones-Thornton-Taylor (JTT) aa matrix and all sites within the MEGA5 program [32]. Node values represent percent bootstrap confidence derived from 2,000 replicates. The molecule and the common species name are followed by the accession number. The new sequences reported in this review are shaded.

To confirm the relationship among the SOCS family members, an unrooted maximum likelihood (ML) tree (Figure 3) was inferred using MEGA software [32] with the Jones-Thornton-Taylor (JTT) aa matrix and all sites. The tree contained all the known full-length fish SOCS molecules and selected tetrapod SOCS members. As seen in the ML tree, all the fish CISHa and CISHb grouped together to form an independent clade and grouped with tetrapod CISH molecules with high bootstrap support (90%), suggesting that the fish CISHa and CISHb/SOCS8 are indeed paralogues of tetrapod CISH.

**Figure 4:**
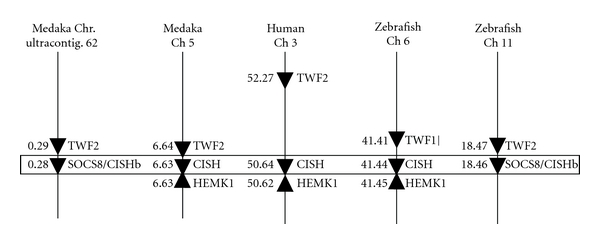
Synteny analysis of the chromosome loci harbouring fish CISHa and CISHb/SOCS8 and mammalian CISH. The analysis was carried out using the synteny browser Genomicus [34] (at the website http://www.dyogen.ens.fr/genomicus-62.02).

Synteny analysis of the chromosomal loci harbouring the CISH and SOCS8 genes in fish and mammalian CISH was carried out using the new synteny browser Genomicus [34] at the website (http://www.dyogen.ens.fr/genomicus-62.02). A TWF2- (twinfilin, actin-binding protein, homolog 2-) like gene is closely linked with both CISH and SOCS8 genes in both zebrafish and medaka, with the same transcriptional direction. The TWF2 gene is also closely linked to CISH in human Ch 2 (Figure 4). In addition, a HEMK1 gene is also closely linked and with the reverse transcriptional direction to CISHs in human Ch 2, medaka Ch 5, and zebrafish Ch6.

Thus, the similar domain structure, conserved gene organisation, ML phylogenetic tree analysis, as well as synteny analysis, suggest that the fish CISH and SOCS8 molecules described by Jin et al. in model fish are indeed paralogues of tetrapod CISH.

### 2.2. Fish SOCS2

Fish SOCS2 genes have been reported in rainbow trout [35] and in the five model fish with only a single gene described in each fish species [20]. A grass carp SOCS2 sequence has also been submitted to Genbank. The SOCS2 and CISH share greater sequence homology with each other than with other members. They also both have three-exon/two-intron gene organisations although in the SOCS2 gene only the last two exons encode for its protein (Figure 1). The existence of two fish CISH paralogues prompted us to look into the possibility that SOCS2 paralogues may exist in salmonids and resulted in the cloning of two additional SOCS2 related cDNAs named SOCS2b and SOCS2b related (SOCS2bRel), respectively, in rainbow trout. The trout SOCS2 molecule reported previously is called SOCS2a hereafter.

Search of the EST database identified two trout ESTs (acc. nos. CX719528 and BX081159) that could be transcribed from SOCS2 related genes. Primers were designed at the putative 5′-UTR and used for 3′-RACE using SMART cDNA prepared from bacterial challenged gill tissues as described previously [23, 24]. The 3′-RACE product amplified using primers against EST CX719528 was 2,521 bp and contained a 5′-UTR of 304 bp, an ORF of 657 bp encoding for 218 aa (designated as SOCS2b, acc. no. FR874096), and a 3′-UTR of 1560 bp. There were two mRNA instability motifs (ATTTA) and a poly A signal, 22 bp upstream of the poly A tail, in the 3′-UTR. The 3′-RACE product amplified using primers against EST BX081159 was 1,581 bp and contained a 5′-UTR of 372 bp, a potential ORF of 333 bp encoding for 110 aa (designated as SOCS-2bRel, acc. no. FR874097), and a 3′-UTR of 876 bp. There were also two ATTTA motifs and a poly A signal, 18 bp upstream of the poly A tail, in the 3′-UTR. It is worth noting that there are six ATTTA motifs in the trout SOCS2a cDNA sequence [35].

**Figure 5:**
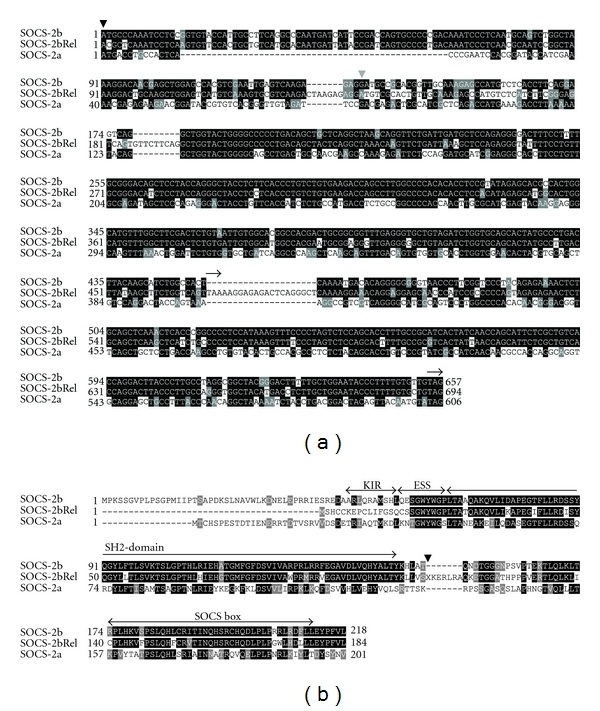
Comparison of the nucleotide (a) and amino acid (b) sequences of the three trout SOCS2 paralogues. (a) The ORFs of trout SOCS2a and 2b as well as a putative ancestral ORF of SOCS2bRel were aligned by ClustalW2 and box shaded. The start codons in SOCS2a and 2b are indicated by ▾ and in SOCS2bRel by grey triangles. The stop codons are indicated by *→*. (b) The three trout SOCS-2 paralogues were aligned using the ClustalW2 program and box shaded. Dashes (–) indicate gaps in the alignment. Note that the translation after the stop codon (indicated by X) that may represent the ancestral SOCS2bRel was also used in the alignment. The putative KIR, ESS region, SH2 domain, and SOCS box domains are indicated above the alignment.

The potential SOCS2bRel ORF was short; however, an ancestral ORF can be constructed by comparison of the three SOCS2 sequences. As shown in Figure 5(a), the ancestral start codon was mutated in SOCS2bRel, and a stop codon was also introduced likely by an insertion just after the sequence encoding for the SH2 domain. The sequence after the stop codon encoding for the putative SOCS box in SOCS2bRel is well conserved, both at the DNA and amino acid sequence levels (Figures 5(a) and 5(b)). However, at the full-length cDNA level trout SOCS2bRel only shared 40.1% and 46.6% identities to SOCS2a and 2b, respectively, whilst the putative ORF of the ancestral SOCS2bRel shared 49.9% and 82.9% identities to SOCS2a and 2b, respectively.

Trout SOCS2a and 2b have similar domain structures to other SOCS2 proteins (Figure 1(b)). The SH2 domain and SOCS box are well conserved (Figure 5(b)). The ESS region is also somewhat conserved, but the KIR and upstream N-terminal have diverged between these two molecules. Due to mutations, the trout SOCS2bRel has a potential ORF that if translated encodes for only a single SH2 domain without the KIR, ESS, and the SOCS box. The SH2 domain shared 51.8% and 88.0% identities to that of SOCS2a and SOCS2b, respectively. As the SH2 domain is critical for phosphotyrosine binding [26], it is possible that the trout SOCS2bRel could function as a potential negative regulator of other SOCS proteins by competition for phosphotyrosine binding.

Trout SOCS2a showed higher identities to other fish SOCS2 molecules (73.6–79.1%) than to tetrapod SOCS2 molecules (47.3–55.8%) (Table 2). However, trout SOCS2b showed similar and lower identities to SOCS2 molecules from both fish (40.8–42.5%) and tetrapods (38.5–41.3%). Trout SOCS2bRel showed the highest identity to trout SOCS2b, with 38.5% overall identity, and 58.7% identity if the putative ancestral gene (including the translation after the stop codon, as in Figure 5) was used, and 88% identity in the SH2 domain. Without the SOCS box, trout SOCS2bRel only showed 23.9–25.9% identities to SOCS2 molecules from other fish species and tetrapods (Table 2).

In the ML phylogenetic tree (Figure 3), trout SOCS2a grouped with other fish SOCS2 molecules and formed an independent clad and again grouped with tetrapod SOCS2 with high bootstrap value (86%) support. In line with the homology analysis (Table 2), trout SOCS2b and 2bRel form an independent clad, but still grouped with the other SOCS2 members. The bootstrap value will perhaps be increased if SOCS2b molecules are discovered in other fish species.

### 2.3. Fish SOCS1 and SOCS3

SOCS1 and 3 share greater sequence homology and other features with each other than with other SOCS family members. Both genes have a two-exon/one-intron structure, but only the last exon encodes for their proteins (Figure 1). Both SOCS1 and 3 have a similar domain structure including a well-defined KIR region which acts as a pseudosubstrate for JAKs, inhibiting JAK kinase activity and having important roles in regulating innate and adaptive immune responses [36].

Fish SOCS1 genes have to date been reported in the five model fish [20, 37] and in rainbow trout [35], with only a single gene described in each species. A grass carp SOCS1 sequence has also been deposited in Genbank. In the ML phylogenetic tree analysis, all of the fish SOCS1 molecules group together and group with the tetrapod SOCS1 with a high bootstrap value (99%), confirming their identities.

Fish SOCS3 genes have also been reported in a number of fish species including the five model fish, as well as in rainbow trout, common carp (*Cyprinus carpio*), and turbot (*Scophthalmus maximus*) [20, 35, 38–40]. SOCS3 sequences from grass carp and catfish (*Ictalurus punctatus*) have also been submitted to the database. It appears that there are two paralogues of tetrapod SOCS3 in fish, with fish SOCS3a molecules having higher identities (48.2–66.2%) to tetrapod SOCS3. Fish SOCS3b molecules share comparable but lower identities to both tetrapod SOCS3 (45.0–52.3%) and fish SOCS3a (42.0–54.8%). In the ML phylogenetic tree, fish SOCS3 paralogues are grouped with tetrapod SOCS3 with high bootstrap value (99%) support (Figure 3).

### 2.4. Fish SOCS4 to 7

SOCS4 to 7 form a subgroup within the SOCS family because of their extended N-terminal regions and have previously been termed the Type I SOCS subfamily [20]. Thus, the N-terminal (excluding the ESS) of human SOCS4, 5, 6, and 7 is 270 aa, 368 aa, 369 aa, and 385 aa, respectively. SOCS4 to 6 all have a two-exon/one-intron structure with only the last exon encoding for the protein. However, SOCS7 has a ten-exon/nine-intron gene organisation (Figure 1). The fish SOCS4, 6, and 7 will be described in this section, whilst SOCS5 along with fish SOCS9 will be discussed in the next section.

**Figure 6:**
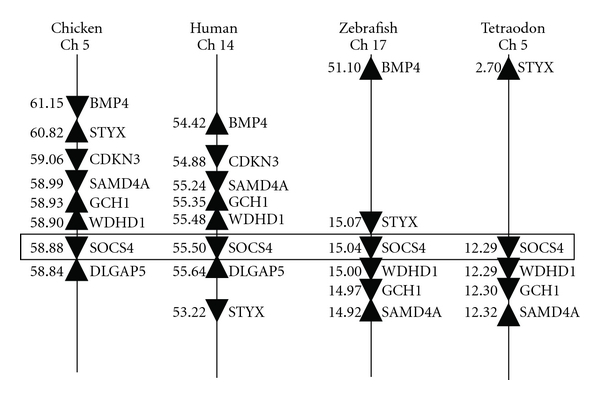
Synteny analysis of the chromosome loci harbouring fish and tetrapod SOCS4. The analysis was carried out using the synteny browser Genomicus [34] (at the website http://www.dyogen.ens.fr/genomicus-62.02).

Fish SOCS4 genes have only been described in the five model fish species [20] and share 40.9–45.9% identities to tetrapod SOCS4. They are apparently related to tetrapod SOCS4, as supported by conserved gene synteny (Figure 6). However, the relationship is complicated by the clustering of the fish SOCS4 molecules to the base of the group of the tetrapod SOCS4 and SOCS5 clades in ML phylogenetic tree analysis (Figure 3).

Fish SOCS6 and 7 genes have also been reported in the five model fish species and in rainbow trout [20, 21]. Two zebrafish SOCS6 sequences exist in the database and share 68.0% identity at the aa level. Fish SOCS6 share 57.6–72.8% identities to tetrapod SOCS6, whilst fish SOCS7 has lower identities of 36.9–52.8% to tetrapod SOCS7. In the ML phylogenetic tree, fish SOCS6 and SOCS7 group with the tetrapod SOCS6 and SOCS7, respectively, and together form a subgroup within the Type I subfamily of SOCS molecules (Figure 3).

### 2.5. Fish SOCS5 and SOCS9

Fish SOCS5 genes have been reported in the five model fish species [20]. Two zebrafish SOCS5 sequences exist that share 62.8% identity. The fish SOCS5 share high amino acid sequence identities (60.1–65.9%, Table 3) to tetrapod SOCS5. SOCS5 related sequences, termed SOCS9 by Jin et al. [20], have also been described in the five model fish, as well as in rainbow trout [21]. Multiple alignment of fish SOCS5, SOCS9, and tetrapod SOCS5 revealed that the C-terminal domain containing the SH2 and SOCS box domains are highly conserved among all the SOCS5 and SOCS9 molecules, whilst SOCS5-specific and SOCS9-specific features can be seen at the N-terminus [21]. However, fish SOCS9 share comparable identities to SOCS5 molecules from tetrapod (42.1–46.2%) and fish (40.7–44.5%, Table 3). From the evidence given below, we reclassify the fish SOCS5 as SOCS5a and fish SOCS9 as SOCS5b to refer to the fact that both fish genes appear to be paralogues of tetrapod SOCS5.

**Figure 7:**
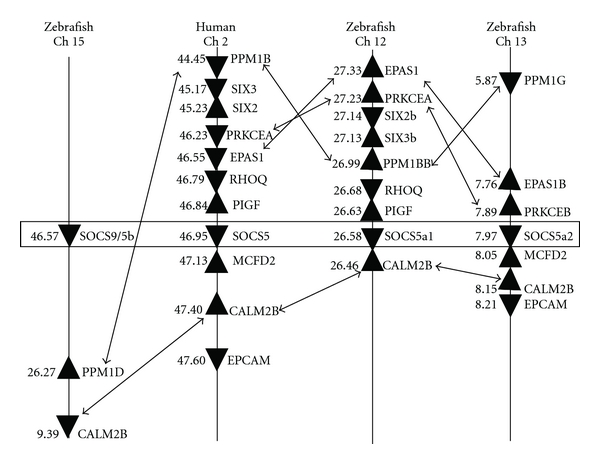
Synteny analysis of the chromosome loci harbouring zebrafish SOCS5a1, 5a2, 5b, and human SOCS5. The analysis was carried out using the synteny browser Genomicus [34] (at the website http://www.dyogen.ens.fr/genomicus-62.02).

The zebrafish SOCS5a1, a2, and b are situated on Chs 12, 13, and 15, respectively, whilst human SOCS5 on Ch 2. The SOCS5 neighbouring genes CALM2B and PPM1 as well as EPAS1 and PRKCEA are syntenically conserved on human Ch 2, and zebrafish Chs 12 and 13, with CALM2B and PPM1 also conserved on zebrafish Ch 15 (Figure 7), suggesting that the zebrafish SOCS5 molecules are indeed paralogues of tetrapod SOCS5. This notion is further supported by ML phylogenetic tree analysis (Figure 3) where the fish SOCS5a and 5b form independent clades, with fish SOCS5a and tetrapod SOCS5 grouping first, with all the SOCS5 molecules grouping together with high bootstrap value (97%) support.

The N-terminal regions of SOCS proteins are variable in length and in aa sequence and are predicted to be disordered. The N-terminal regions of SOCS5–7 are longer than SOCS1–3 and CISH, while the SOCS4 N-terminal region is of intermediate length [41]. Although the function of the remaining N-terminal sequence among the SOCS family members remains undefined, it could affect the SH2 domain binding to its target. SOCS5 can inhibit IL-4, IL-6, and leukemia inhibitory factor-induced signaling, as well as EGFR signaling. The expression of SOCS5 led to a marked reduction in expression levels by promoting EGFR degradation in a ligand-independent manner [42]. Thus, the divergent N-terminal in fish SOCS5 paralogues may allow different target binding.

## 3. Fish SOCS Gene Expression and Modulation

### 3.1. Constitutive Expression

The constitutive expression of fish SOCS genes in healthy individuals has only been systematically examined in tetraodon and rainbow trout, with the exception of SOCS3 expression that is also investigated in common carp and turbot.

The expression of trout CISHa and SOCS1, 2, 3, 5b, 6, and 7 has been examined in eight tissues from six healthy trout, three mucosal sites (gills, skin, and intestine), three internal fish immune tissues (liver, spleen, and head kidney), and muscle and brain, by real-time PCR. It is worth noting that the cephalic portion of fish kidney (head kidney or pronephros) is a central immune organ considered analogous to mammalian bone marrow. The expression of all seven SOCS genes is detectable in all the tissues examined, with the liver expressing the lowest levels of all genes except CISHa [21, 35]. The brain expresses the highest levels of most SOCS genes except SOCS1 and SOCS3. The highest expression of trout SOCS1 is in the intestine whilst for SOCS3 it is the gills. In general, CISHa, SOCS1, and 3 are highly expressed in most of the tissues examined, especially in the immune-related tissues (intestine, head kidney, spleen, gills, and skin) [21, 35].

The expression of SOCS genes in tetraodon shows some differences to that seen in trout. Tetraodon SOCS1 is highly expressed in liver, gonad, and spleen tissues and moderately in intestine, gills, and kidney [37], whilst in trout SOCS1 is most highly expressed in intestine followed by spleen, head kidney, and gills [35]. The tetraodon SOCS5b (SOCS9) is highly expressed in head kidney and spleen, whilst the trout SOCS5b is only expressed at a moderate level in these tissues [21]. SOCS3 expression has been examined in tetraodon, trout, common carp, and turbot with a consensus that the gills express the highest level, with high levels also present in immune-related tissues, including head kidney, spleen, and intestine [35, 38–40].

The expression of the zebrafish SOCS1 and carp SOCS3 genes has also been examined during ontogeny. Zebrafish SOCS1 is highly expressed in eggs, and during embryonic development its transcripts are evident at the one-cell stage and continue to be uniformly distributed until the gastrula period when the expression becomes predominantly confined to the mesoderm [43]. In contrast, the carp SOCS3 transcript is not detectable in sperm and eggs, and first appears at 4 h after fertilization, after which it gradually increases up to 4 weeks after fertilization [39].

### 3.2. Modulation of SOCS Gene Expression by Fish Cytokines and Immune Stimulants

Most mammalian SOCS proteins are induced by cytokines and therefore act in a classical negative-feedback loop to inhibit cytokine signal transduction. With our recent success in the production of bioactive recombinant trout cytokines, trout SOCS gene expression in response to IFN-*γ*, IL-1*β*, IL-6, and IL-21 has been investigated. It is apparent that the induction of SOCS gene expression is SOCS member, cell type, and cytokine dependent.

Both trout IFN-*γ* and IL-1*β* upregulate the expression of SOCS1, 2, and 3 in the fibroid cell line RTG-2, but only IFN-*γ* can upregulate these three genes in the monocyte/macrophage-like cell line RTS-11 [35]. However, neither of these two cytokines have any significant effects on the expression of CISHa, SOCS5b, 6, and 7, in RTG-2 or RTS-11 cells [21].

Trout IL-6 can rapidly induce SOCS1, 2, 3, and CISHa expression in RTS-11 cells. Whilst SOCS3 expression is rapidly increased and peaks after just 0.5 h of IL-6 stimulation, with a 15-fold increase over the controls, the expression of SOCS1, 2, and CISHa peaks somewhat later at 2 h after IL-6 stimulation. The expression of SOCS genes comes down quickly from the peak increase, and by 6 h post-treatment there are no significant differences in SOCS expression between the IL-6 stimulated and unstimulated cells [8].

Trout SOCS1 and 3 have also been shown to be upregulated by IL-21 in head kidney leucocytes [12], and turbot SOCS3 expression is increased by TNF-*α* in head kidney macrophages [40].

The expression of fish SOCS genes in response to pathogen-associated molecular patterns (PAMPs) such as LPS and poly I:C has also been examined in a few studies. Jin et al. [20, 37, 38] reported that *in vivo *injection of LPS induces the expression of tetraodon CISHa and SOCS1 to 5 in head kidney at 12 h after exposure. However, the expression of CISHb and SOCS6 is not affected by LPS injection. Tetraodon SOCS3 mRNA level is also increased in gill, spleen, intestine, skin, liver, and heart after LPS stimulation [38]. LPS upregulates the expression of trout SOCS1, 2, and 3 in RTS-11 cells in a time-dependent manner but has no effect on the expression of CISHa, SOCS5b, 6 and 7. In primary trout splenocyte cultures, LPS significantly increases the expression of CISHa, SOCS1 and SOCS2 24 h after stimulation, with SOCS1 also increased at 4 h and 8 h after stimulation. A significant decrease in expression of the other SOCS genes is also seen at the early time points relative to unstimulated cells [21].

Poly I:C is a strong stimulator of SOCS1 expression in RTS-11 cells and also significantly upregulates the expression of SOCS2 and 3 at later time points, but has no effect on CISHa and SOCS6 and significantly downregulates the expression of SOCS5b and SOCS7. Curiously, poly I:C stimulation has no effect on splenocyte SOCS gene expression [21].

PMA (phorbol-12-myristate-13-acetate) is a protein kinase C activator and can stimulate the expression of cytokines and other immune-relevant genes. PMA at 0.5–500 ng/mL increases the expression of SOCS1, 2, and 3 but has no effects on CISHa, SOCS5b, 6, and 7 in RTG-2 cells. The modulation of SOCS gene expression by PMA in RTS-11 cells is more widespread and also apparently more dose dependent. As with RTG-2 cells, the expression of SOCS1, 2 and 3 is significantly upregulated at the lowest (0.5 ng/mL) and highest (500 ng/mL) doses used, but is significantly downregulated by incubation with 5 ng/mL. PMA can also increase the expression of SOCS5b and 6 at some doses but has no effects on the expression of CISHa and SOCS7 [21].

### 3.3. Modulation of SOCS Gene Expression by Viral Infection

The response of fish SOCS gene expression to viral infection has only been reported in common carp to date. Spring viraemia of carp virus (SVCV) infection increases carp SOCS3 expression from day 1 in intestine and day 3 in gills and thymus and maintains a high level of expression until day 10 [39]. SVCV also upregulates carp SOCS3 expression at day 3 and day 5 in head kidney and kidney and at day 5 and day 7 in spleen but loses its effects thereafter.

To gain more insight into the role of fish SOCS genes in antiviral defence, we have investigated SOCS gene expression in brown trout (*Salmo trutta*) after viral hemorrhagic septicemia virus (VHSV) infection. VHSV is a negative single-stranded RNA virus that causes a serious systemic viral hemorrhagic septicemia in a wide variety of wild and cultured fish species and has been one of the major threats to the development of salmonid aquaculture [44, 45]. A VHSV genotype I sublineage a (J167) was isolated from a trout farm in Yorkshire, UK in 2006 [46]. The virus was propagated and titrated in EPC cells at 15°C. For infection of brown trout, a salmonid that is native in Europe and has variable disease resistance relative to the more commonly studied rainbow trout [47, 48], the fish were grown in pathogen-free water conditions and bath challenged for 4 h at 12°C using a suspension of VHSV (5.6 × 10^5^ TCID50/mL). At the same time a control group was mock-exposed to the same sterile transport medium (Glasgow minimum essential medium, SAFC Biosciences) used to suspend the virus. Kidney samples were subsequently collected and the presence of VHSV assayed on EPC cells at 15°C, with a clear CPE present within 1–3 days of exposure. Only 6.7% of fish were VHSV positive at 1 day postinfection (pi), but 76.7% of fish were VHSV positive after 3 days of exposure. Infected fish showed the typical clinical signs of VHS, with the first mortalities recorded after 8 days pi. To correlate the SOCS gene expression with the success of viral infection, three groups, each of 5 fish, were chosen for gene expression analysis in the kidney. One group was the unexposed control fish that were negative for VHSV. The second group contained exposed fish that were negative for VHSV in the kidney at 3 days pi, and the last group was positive for VHSV at this time, suggesting a successful viral infection.

**Figure 8:**
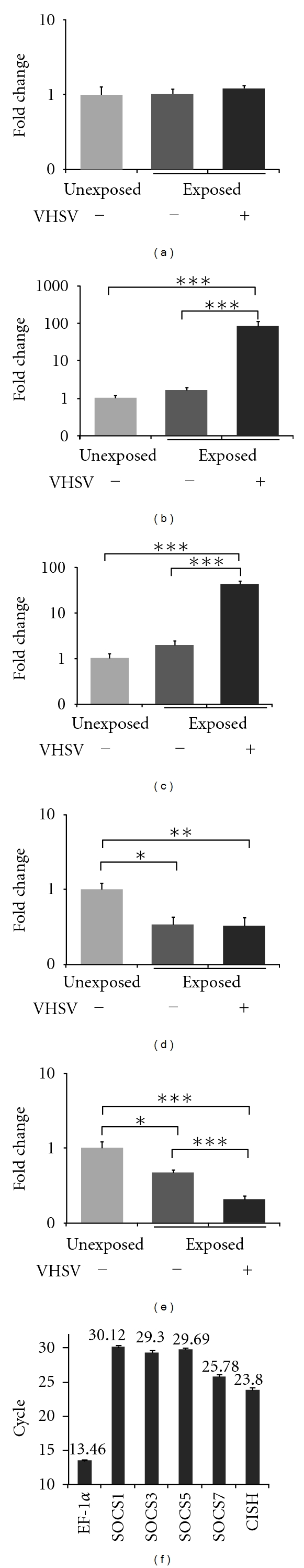
Modulation of SOCS gene expression in brown trout by VHSV infection. Naïve brown trout were bath challenged for 4 h at 12°C in a suspension of VHSV (isolate J167) at 5.6 × 10^5^ TCID50/mL, or the sterile medium used for suspension of the virus as control. Kidney tissue was collected at 3 days postinfection for total RNA preparation and viral burden assessment, which revealed that 76.7% of fish were VHSV positive after exposure but negative in the control group. Thus, the expression of CISH (a), SOCS1 (b), SOCS3 (c), SOCS5 (d) and SOCS7 (e) was examined by real-time reverse-transcription (RT) PCR in three groups: the unexposed controls, the VHSV-detectable, and VHSV-undetectable challenged fish as described previously [21, 35]. Briefly, tissues were collected and stored in RNAlater (Ambion) or directly used for total RNA isolation using Trizol (Invitrogen). The resulting total RNA was converted into cDNA and quantified by real-time PCRs using a LightCycler 480 system (Roche). The PCRs were performed in duplicate for each sample, and transcript level was calculated using the quantitative fit points method in the integrated LightCycler 480 software. The gene expression was first normalised to that of the housekeeping gene EF-1*α* and expressed as a fold change relative to the unexposed control. The results are presented as mean + SEM from 5 fish. The *P* value of an LSD post hoc test after a significant one-way analysis of variance between the VHSV exposed and control fish is shown above the bars as *P < 0.05, **P ≤ 0.01, and ***P ≤ 0.001. For comparison of the relative expression levels of genes examined, the cp values (the crossing point at which the fluorescence crosses the threshold) in the real-time PCR in 10 unexposed fish are presented as means ± SD (f). Please note that the higher the cp value, the lower the expression level.

**Figure 9:**
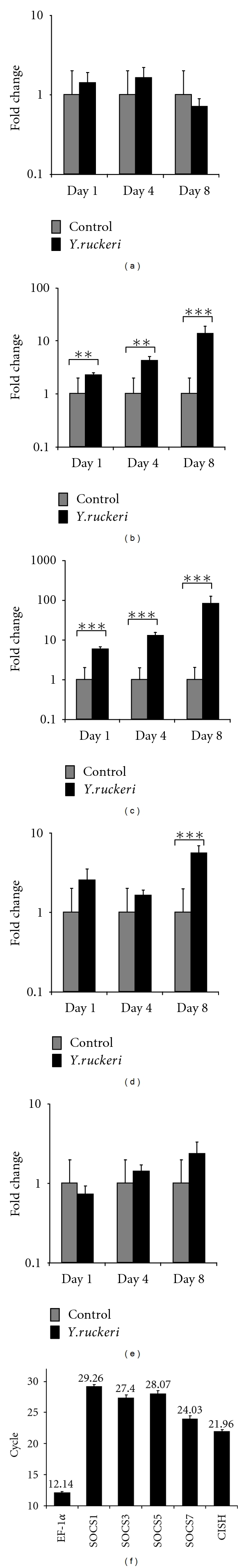
Modulation of SOCS gene expression in brown trout by *Yersinia ruckeri* infection. Naïve brown trout were exposed for 4 h at 16°C by bath challenge in a suspension of *Yersinia ruckeri* (isolate 06041) at a concentration of 2.7 × 10^7^ cfu/mL, as described previously [49]. At the same time a control group of fish was exposed to the same dilution of sterile medium used to suspend the bacteria. Fish from each group were sampled at 1, 4, and 8 days postinfection. Kidney swab screenings demonstrated the exclusive presence of *Y. ruckeri *in the pathogen-challenged fish but not in control fish, The expression of CISH (a), SOCS1 (b), SOCS3 (c), SOCS5 (d), and SOCS7 (e) was examined in kidney samples by real-time RT-PCR as described in Figure 8. The gene expression was first normalised to that of the housekeeping gene EF-1*α* and expressed as a fold change relative to the unexposed control. The results are presented as mean + SEM. The *P* value of an LSD post hoc test after a significant one-way analysis of variance between the VHSV exposed and control fish is shown above the bars as **P ≤ 0.01 and ***P ≤ 0.001. For comparison of the relative expression levels of genes examined, the cp values in the real-time PCR in 15 unexposed fish are presented as means ± SD (f). Please note that the higher the cp value, the lower the expression level.

**Figure 10:**
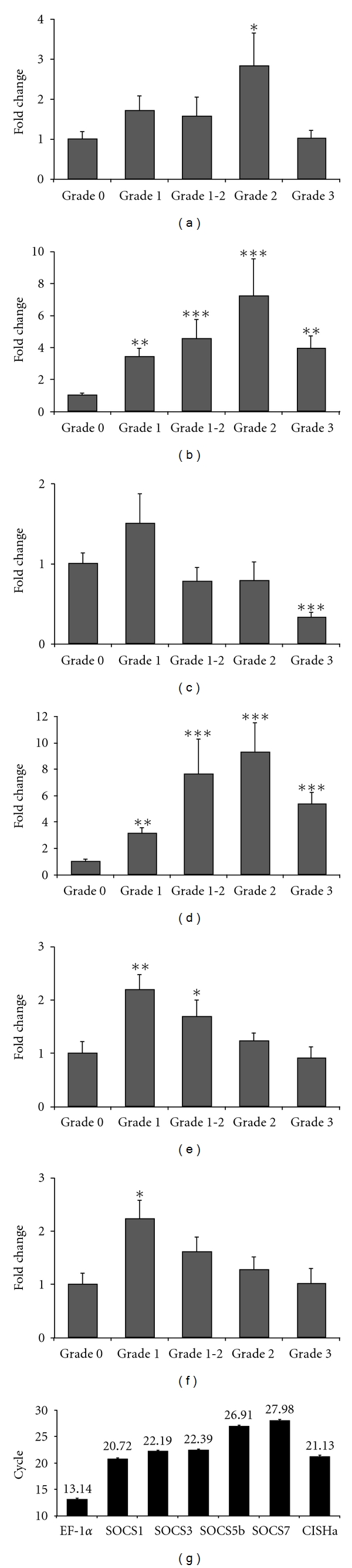
Modulation of SOCS gene expression in rainbow trout by PKD infection. Rainbow trout (50–100 g) were sampled in the summer of 2001 during a natural outbreak of PKD on a fish farm in Hampshire (UK). The fish exhibited clinical pathology and kidneys graded from 1 to 3 on the swelling grade. Approximately 100 mg of kidney tissue was removed from an area of the posterior kidney immediately below the dorsal fin, an area of the kidney normally associated with the onset of pathology [50] and stored in RNAlater (Ambion) at −80°C. A naïve unexposed control group (grade 0), from the same egg source, was sampled from a farm with no history of PKD infection. Routine checks for other parasite infestations and opportunistic bacterial pathogens presence were negative. Following total RNA extraction, 48 individual cDNAs were generated for real-time RT-PCR analysis of the expression of CISHa (a), SOCS1 (b), SOCS2 (c), SOCS3 (d), SOCS5 (e), and SOCS7 (f), as described in Figure 8. The gene expression was first normalised to that of the housekeeping gene EF-1*α* and expressed as a fold change relative to the grade 0 fish. The results are presented as mean + SEM (*n* = 13, grade 0; 6, grade 1; 9, grade 1-2; 10, grade 2; 10, grade 3). The *P* value of an LSD post hoc test after a significant one-way analysis of variance between the PKD exposed groups and grade 0 control fish is shown above the bars as *P < 0.05, **P ≤ 0.01, and ***P ≤ 0.001. For comparison of the relative expression levels of genes examined, the cp values in the real-time PCR in the kidney of 13 unexposed grade 0 fish are presented as means ± SD (g). Please note that the higher the cp value, the lower the expression level.

Total RNA was extracted from the kidney tissues and converted to cDNA. The real-time RT-PCR assay was as described previously [21, 35], using primers designed for rainbow trout [21, 35] or based on brown trout SOCS1 and 3 sequences (acc. nos. FR873839, FR873840, and FR873841). It is worth noting that the constitutive expression of SOCS1 and 3 in the kidney of brown trout (delta Ct is 17.12 for SOCS1 and 15.26 for SOCS3, Figure 9(f)) is much lower than that in rainbow trout (delta Ct relative to EF-1*α* is 7.58 for SOCS1 and 9.25 for SOCS3, Figure 10(g)). The expression of SOCS1, 3, and 5 in kidney was low compared to that of CISHa and SOCS7 in the control unexposed fish (Figure 8(f)). CISH expression was not affected by VHSV infection (Figure 8(a)). The expression of SOCS5 and 7 was downregulated by VHSV exposure (Figures 8(d) and 8(e)), with SOCS7 expression lower in VHSV-positive fish compared to VHSV-negative fish. The expression of brown trout SOCS1 and 3 between the unexposed and VHSV-negative exposed fish was not significantly different, but in VHSV-positive fish their expression was significantly upregulated (SOCS1 up to 84-fold, SOCS3 up to 43-fold, Figures 8(b) and 8(c)). It will be interesting to study in future experiments whether this may be one facet of the evasion mechanisms of the virus.

### 3.4. Modulation of SOCS Gene Expression by Bacterial Infection

Bacterial infection induces the expression of SOCS genes that may counteract the host immune defence to promote bacterial survival. For example, one-third of humans carry *Mycobacterium tuberculosis*, the etiological agent of tuberculosis where microbe/host immune response interactions result in persistent or active tuberculosis. Despite the concomitant heightened levels of Th1-type mediators, the expression of SOCS1 and 3 is highly upregulated in tuberculosis patients. The effect of overexpression of SOCS genes could limit the proliferation and expansion of Th1 cells, as well as responsiveness to Th1-type cytokines, and, in turn, less production of Th1-type cytokines resulting in promotion of *M. tuberculosis* survival [51].

Fish SOCS gene expression in response to fish bacterial pathogens has been investigated in two reports. Zhang et al. [40] reported that the fish pathogen *Listonella anguillarum,* one of the most important causative agents of vibriosis in fish and shellfish around the world, stimulates turbot SOCS3 transcription in kidney, spleen, liver, and gill in a time-dependent manner. In kidney and gill, significant induction of SOCS3 is detected from 12 h to 48 h pi and peaks at 24 h pi, while in liver SOCS3 induction reaches significant levels at 12 h and 24 h pi, with a peak level at 12 h pi. In contrast, in spleen significantly upregulated SOCS3 expression was seen as early as 4 h and 8 h pi, to 24 h pi [40].

We have previously reported on SOCS gene expression after *Yersinia ruckeri* infection of rainbow trout. *Y. ruckeri* is a Gram-negative ovoid or rod-shaped bacterium that is the aetiological agent of yersiniosis or enteric redmouth disease (ERM) that causes significant economic losses in salmonid aquaculture worldwide [52]. In some cases the losses due to this disease can reach 30–70% of the stock. Despite the importance of ERM and the existence of a successful vaccine, little is known about the precise pathogenic mechanisms by which *Y. ruckeri* causes disease [52]. The injection of *Y. ruckeri* (strain MT3072) into rainbow trout results in high mortality after 3-days. The expression of all the SOCS genes is significantly upregulated 2 days pi compared to 1 day pi, even in the saline-injected control fish, suggesting that operational stressors (i.e., injection, netting, etc.) can modulate SOCS gene expression. However, only the expression of CISH and SOCS1 and 3 was specifically upregulated by this bacterial infection [21].

Here we have also investigated SOCS gene expression in *Y. ruckeri*-infected brown trout. For this study, *Y. ruckeri* isolate 06041 was used, a strain that causes high mortality in rainbow trout and Atlantic salmon in the UK [49]. Naïve brown trout were grown in pathogen-free water conditions and exposed for 4 h to an experimental bath challenge in 30 L tanks at a final concentration of 2.7 × 10^7^ cfu/mL at 16°C as described previously [49]. At the same time control fish were mock-challenged using the same saline (Dulbecco's phosphate buffered saline, Sigma) used to suspend the bacteria. Kidney tissue was dissected at 1, 4, and 8 days pi and stored in RNAlater at −80°C for subsequent total RNA extraction and cDNA synthesis. Kidney swabs demonstrated the presence of *Y. ruckeri* in the pathogen-challenged fish but not in control fish by a monoclonal antibody agglutination test (using Mono-Yr, Bionor, Norway). As seen in rainbow trout, the expression of brown trout SOCS1 and 3 was significantly increased in a time-dependent manner from day 1 to day 8 pi (SOCS1 up to 14-fold, and SOCS3 up to 80-fold, Figures 9(b) and 9(c)). SOCS5b expression was also increased at day 8 pi. In contrast to rainbow trout, brown trout CISH expression was not changed upon *Y. ruckeri* infection compared to control fish (Figure 9(a)).

### 3.5. Modulation of SOCS Gene Expression by Parasite Infection

Parasitic infections can also induce SOCS gene expression, and this may contribute to the parasite's immune evasion strategies. For example, the protozoan parasite *Leishmania donovani*, the causative agent of visceral leishmaniasis, induces SOCS3 expression that results in a potent inhibitory mechanism to suppress macrophage activation and interfere with the host immune response [53]. The intracellular parasite *Toxoplasma gondii *that survives and multiplies in professional phagocytes such as macrophages induces SOCS1 expression that inhibits IFN-*γ* receptor signaling [54]. Little is known about the role of SOCS gene expression in fish parasitic infections. Thus, here SOCS gene expression in a natural outbreak of proliferative kidney disease (PKD) in rainbow trout has been examined.

PKD is an economically important disease of farmed and wild trout populations in Europe and North America [55, 56]. The aetiological agent is a myxozoan parasite, *Tetracapsuloides bryosalmonae *(Myxozoa: Malacosporea) [57], now known to be a cnidarian [58]. It has a two-host life cycle, involving both vertebrate (salmonid fish) and invertebrate (*Fredericella sultana,* a freshwater bryozoan) hosts. Spores released from infected bryozoans float in the water where they can infect the host fish [59, 60]. The myxozoan histozoic proliferation induces a massive lymphocyte hyperplasia in the interstitial tissue, resulting in granulomatous lesions and massive swelling of spleen and kidney [61]. Normally, clinical symptoms of the disease appear after 6–8 weeks pi [55, 62]. In severe outbreaks, the disease causes high mortalities, up to 90%, due to anaemia, stress, and secondary bacterial infections. The severity of clinical signs during PKD can be monitored using a kidney-swelling index that is divided into five grades, from 0 to 4. [62]. Grade 0 corresponds to healthy, unaffected kidneys, while grade 3 kidneys have extreme swelling associated with the most severe clinical signs, and grade 4 is the terminal stage of the disease [62].

Rainbow trout 50–100 g were sampled during a natural outbreak of PKD in the summer of 2001 from a fish farm in Hampshire (UK), when fish exhibited clinical pathology, with kidney-swelling grade ranging from 1 to 3. Approximately 100 mg of kidney tissue was removed from an area of the posterior kidney immediately below the dorsal fin, an area of the kidney normally associated with the onset of pathology [50] and stored in RNAlater (Ambion) at −80°C. Concurrently, a naïve unexposed control group (grade 0), from the same egg source, was sampled from a farm with no history of PKD infection. Following total RNA extraction, 48 individual cDNAs were generated for real-time PCR analysis of gene expression, as described above, using primers for trout SOCS genes [21, 35]. As a matter of farm routine, checks for other parasite infestations were conducted. With respect to the kidney itself, swabs were taken under aseptic conditions at the time of sampling and streaked onto standard TSA plates (Becton-Dickinson) as a means of checking for the presence of opportunistic bacterial pathogens (e.g., *R. salmoninarum* and *Aeromonas salmonicida*). Streaked plates were incubated at 20–22°C for 48 h and examined for any bacterial growth. As shown in Figure 10, the kidney expression of rainbow trout SOCS1 and 3 was significantly upregulated and increased with increasing pathology up to grade 2 fish. Moderate upregulation (2-fold) of SOCS5b and 7 was also seen in kidneys with low pathology (grades 1 and 1-2), although no upregulation was observed in more advanced disease states (Figures 10(e) and 10(f)). Lastly, modest upregulation of CISHa was seen in grade 2 fish, whilst SOCS2 expression was significantly downregulated in grade 3 fish that had severe clinical signs. Agar plates prepared from kidney swabs did not reveal the presence of other microbial pathogens which suggests that the observed gene upregulation is due to the presence of *T. bryosalmonae*.

It is known that PKD preferentially induces the expression of IFN-*γ*, T-bet, and IL-2, markers for mammalian Th1-cell development/responses, with expression of proinflammatory genes such as IL-1*β* being unaltered [63]. As both SOCS1 and 3 downregulate IFN-*γ* signaling, their upregulation may represent an evasion strategy of *T. bryosalmonae* to dampen IFN-*γ* signaling and host immune defense.

## 4. Fish SOCS Gene Function

The functional roles of fish SOCS genes have begun to be evaluated. Zebrafish SOCS1 has been shown to interact with both zebrafish JAK2a and STAT5.1 *in vitro* and *in vivo* [43]. In cells cotransfected with hyperactive zebrafish JAK2a and STAT5.1, FLAG-tagged SOCS1 significantly decreases STAT5.1 DNA binding. Morpholino-mediated knockdown of SOCS1 results in perturbation of specific hematopoietic populations, leading to a reduction in the size of the developing thymus later in embryogenesis. Coinjection of STAT5.1 morpholino or the JAK2 inhibitor AG490 significantly reduces the effects of SOCS1 knockdown. These studies demonstrate a conserved role for SOCS1 in T-cell development that is mediated, at least in part, via its effects on receptors using the JAK2-STAT5 pathway [43].

Growth hormone (GH) is a major regulator of postnatal growth in mammals and mediates its effects via signalling through the JAK2/STAT5b and other pathways [64]. GH induces expression of several SOCS family members, including CISH, SOCS1, 2, and 3. Each of these SOCS proteins has been shown to interact with the GH receptor (GHR) and when overexpressed interfere with the JAK2-STAT5b pathway, suggesting that SOCS proteins may regulate GH signaling [26]. GH transgenic carps express higher levels of SOCS3 in the thymus, head kidney, spleen, and intestine but not in gills and kidney [39]. In zebrafish, homozygote individuals of the GH-transgenic lineage F0104 express double the amount of GH compared with hemizygote individuals. However, their growth is slower than the hemizygote individuals and similar to nontransgenic fish. It has been found that the expression of SOCS1 and 3 is significantly higher in the liver of the homozygotes versus hemizygotes and nontransgenic individuals, suggesting that the heightened expression of SOCS1 and 3 in homozygotes downregulates GH signalling [65]. However, the biological role of SOCS1 and 3 under normal physiological conditions warrants further investigation. SOCS proteins are generally lowly expressed and can be induced rapidly by cytokines that are upregulated following immunological insults and physiological stresses. Thus, the higher level of GH expression in homozygotes may present as a physiological stress, whereby a higher energy demand for increased growth results in increased expression of SOCS1 and 3. The resultant downregulation of GH signalling pathways (e.g., JAK2/STAT5b) by SOCSs in GH homozygotes may serve to save energy to deal with this stressful situation. A comparative study on the expression of SOCS genes as well as other stress-induced genes in the homozygotes under conditions of different food availability may shed light on the involvement of a stress response to the effects seen under high GH levels.

Lastly, overexpression of turbot SOCS3 in head kidney macrophages significantly reduced respiratory burst activity, nitric oxide production, bactericidal activity and TNF-*α*, IL-1*β*, and CC-chemokine transcript levels [40]. In contrast, HK macrophages transfected with a mutant SmSOCS3, truncated at the SH2 domain and lacking the SOCS box, exhibited similar levels of respiratory burst activity, nitric oxide production, and bactericidal activity relative to control cells.

## 5. General Discussion

Homologues of all the eight mammalian SOCS family members have been discovered in fish, with many of them having multiple copies in fish. The CISH, SOCS3, and 5 molecules all have duplicates, as found in all the five model fish used for this analysis and that have their genome sequences available to interrogate. Three SOCS2-like genes have been found in rainbow trout and three SOCS5 genes in zebrafish, in addition to two SOCS6 genes in zebrafish, leaving only SOCS1, 4, and 7 that have not yet had duplicates discovered. The duplicated genes may have arisen from gene/genome duplication events in fish. It is now widely accepted that the vertebrate genome experienced two rounds of whole genome duplication (WGD) after the emergence of urochordates and before the radiation of jawed vertebrates (R1 and R2). WGDs seem to have had a crucial role in the emergence of jawed vertebrate adaptive immunity by providing raw genetic materials [66]. Teleost fish experienced a further fish-specific WGD event during their early evolution, some 305–450 million years ago, before the teleost radiation (R3) [67, 68]. In addition a fourth WGD (R4) occurred through an autotetraploidization event in the common ancestor of salmonids between 25 and 100 million years ago [69]. The duplicated genes described in this paper may arise from R3 or R4 WGD (in salmonids). The paralogues of tetrapod CISH, SOCS3, and 5 exist in all the five model fish genomes, suggesting that they arose from R3 WGD. There are three SOCS2 sequences in rainbow trout that can be separated into two subgroups, as SOCS2a and 2b, that may also have arisen from R3 WGD. The trout SOCS2b and 2bRel share high sequence identity and thus may have arisen from the more recent R4 salmonid WGD. However, under specific circumstances local chromosomal duplication or gene duplication events may also contribute to the increase in copy number of genes. For example, the two SOCS5a and two SOCS6 genes in zebrafish may have arisen in this way. Clarification of the origins of paralogues will require genome sequences from more species. In light of the fish-wide 3R WGD and salmonid-wide 4R WGD, it is quite possible that more paralogues of fish SOCS genes have yet to be discovered.

Duplicated genes can acquire a change in function, either via changes in regulatory control or via changes in protein function. In the context of tetraodon SOCS paralogues reported by Jin et al. [20] (i.e., the duplicated CISH and SOCS5 molecules), they are found to be differentially expressed in tissues and differentially modulated by LPS stimulation. For example, CISHa is widely expressed, but CISHb/SOCS8 is only present in a few tissues. Similarly, SOCS5b/SOCS9 is highly expressed in head kidney, spleen, and heart, whilst SOCS5a expression is very low in these tissues. In addition, the expression of CISHa and SOCS5b is highly induced *in vivo* by LPS injection compared to their paralogues. The change of expression and responsiveness to modulation may allow these paralogues to affect a very fine-tuned role in fish.

All SOCS proteins display a three-part architecture (Figure 1). A central SH2 domain is involved in substrate binding through recognition of cognate phosphotyrosine motifs. SOCS proteins can modulate cytokine receptor signaling by multiple complementary mechanisms. First, the KIR found in the N-terminal domain of SOCS1 and 3 inhibits the activity of JAKs by acting as a pseudosubstrate. Second, SOCS proteins can suppress signaling by competing with downstream STATs for binding to shared phosphorylated motifs of the activated receptor. Third, SOCS proteins can regulate signal transduction by linking their substrates to the ubiquitination machinery via the SOCS box leading to its proteasomal degradation [70]. The trout SOCS2bRel molecule only has an SH2 domain without the SOCS box and suggests that this molecule may mainly function as a competitor for phosphotyrosine binding, thus regulating the function of other SOCS proteins. Although the SOCS molecules are generally conserved in the SH2 domain and the SOCS box, the fish SOCS paralogues show great variance at the N-terminal, as seen with the N-terminals of fish CISH (Figure 2) and SOCS5 [21] which are quite divergent. The diversification of the N-terminal of fish SOCS paralogues may modify the binding of the adjacent SH2 domain to its target.

It appears that many immune relevant genes, including many cytokines and their cognate receptors, have been retained after the teleost fish R3 and salmonid R4 WGDs. For example, IL-1*β* [71], TNF-*α* [72, 73], MCSF [24], and many IL-2 family cytokines and their cognate receptors [4, 5] have multiple isoforms in teleost fish. The multiple isoforms of cytokines/receptors are mirrored by multiple copies of negative regulators of cytokine signaling, the SOCS genes, suggesting that a more complicated cytokine network may exist in teleost fish.

Fish SOCS gene expression is under strict control, and different members follow different kinetics in response to the same stimulants. For example, in response to IL-6 stimulation, trout SOCS3 expression peaks within half an hour, whilst SOCS1 and SOCS2 peak later, and no difference is seen after 6 h of stimulation [8]. It is known that some SOCS can cross-modulate other SOCS members. For example, prolactin signaling is suppressed by expression of SOCS1 but is restored by cotransfection of SOCS2 [74]. SOCS2, as well as SOCS6 and 7, can interact with all members of the SOCS family. SOCS2 may thus function as a molecular bridge between a ubiquitin-protein isopeptide ligase complex and SOCS proteins, targeting them for proteasomal degradation [75]. The sequential induction of some SOCS members by cytokines might act to restore cellular sensitivity for subsequent stimulation by suppressing the inhibitory effects of other SOCS proteins [70].

Pathogens can escape from host defence by induction of SOCS expression [47, 53, 54]. The expression of different SOCS members in fish has been shown to be induced by viral, bacterial, and parasitic infection (Figures 8–10), and, in all the cases, SOCS1 and 3 expression is upregulated. Interestingly, the expression of these two SOCS members is much higher in the kidney of rainbow trout compared to that of brown trout. It is worth noting that differences in disease susceptibility exist between these two trout species [51, 52], and it is possible that expression of SOCS genes may function as biomarkers of disease resistance.

Initial functional studies show that fish SOCS affect cytokine signaling via the JAK2-STAT5 pathway [43]. To fully elucidate the function of fish SOCS proteins will be complicated with so many paralogues of SOCS members and cytokines/receptors present. Nevertheless, overexpression of turbot SOCS3 in head kidney macrophages was able to significantly reduce respiratory burst activity, NO production, and bactericidal activity [40]. In mammals, SOCS1 is particularly important for downregulating IFN-*γ* (Th1) effects, whilst SOCS3 inhibits IL-6 (Th17) responses [76, 77]. Thus, inhibition of SOCS gene expression may be a potential target in future studies aimed at modulating T-helper cell development and function, with a view to improving vaccine efficacy, and enhancing disease resistance by dietary means in fish.

## Figures and Tables

**Table 1:** Amino acid identities (top right) and similarities (bottom left) of the fish CISHa and b molecules and CISH molecules from birds, amphibians, and mammals. The accession numbers of the protein sequences are given in Figure 3.

	aa	1	2	3	4	5	6	7	8	9	10	11	12	13	14	15	16	17	18	19	20	21	22
(1) Trout-a	225		90.2	97.8	72.0	65.3	64.9	69.0	64.6	53.2	61.1	57.3	56.9	46.2	43.8	49.6	48.1	48.0	38.6	36.8	38.6	40.7	41.5
(2) Salmon-a1	225	94.2		90.7	71.1	68.4	68.0	72.6	67.7	53.0	60.5	55.5	55.0	47.3	43.8	49.3	47.2	49.3	42.7	40.7	40.9	43.0	43.5
(3) Salmon-a2	225	97.8	94.7		72.4	66.7	65.5	70.4	65.9	52.8	61.6	57.3	56.9	45.4	42.5	48.7	47.6	48.4	39.4	37.5	39.4	41.5	42.7
(4) Smelt-a	220	81.8	82.7	82.2		70.4	68.6	72.1	65.9	57.8	61.0	55.1	55.3	45.9	45.4	48.7	45.6	51.1	42.0	40.8	40.5	42.6	40.8
(5) Tetraodon-a	225	81.8	83.6	83.1	84.4		89.8	84.1	76.5	53.3	57.8	51.3	50.4	43.8	45.5	47.6	43.3	45.3	40.5	40.0	39.8	42.2	42.2
(6) Fugu-a	225	78.7	80.4	80.9	82.7	94.7		83.2	78.3	52.2	55.4	52.3	52.3	44.1	44.8	48.0	42.9	44.3	42.0	42.0	40.3	39.5	41.6
(7) Stickleback-a	226	81.9	84.5	83.2	82.7	92.5	89.4		81.4	53.0	58.8	54.6	53.8	43.4	46.3	47.4	43.0	47.3	43.0	41.4	40.8	41.8	42.7
(8) Medaka-a	226	78.8	80.5	80.1	78.3	86.7	86.3	89.8		51.3	55.3	51.2	51.0	43.6	44.8	43.6	42.4	48.7	41.8	41.5	38.6	41.7	40.9
(9) Zebrafish-a	212	66.2	67.1	66.2	70.5	70.7	70.2	69.0	68.6		71.1	49.6	50.0	43.6	45.3	48.4	47.8	49.5	41.1	39.2	41.3	42.6	41.9
(10) gCarp-a	222	75.1	76.9	76.0	76.6	79.6	75.6	75.7	75.7	79.7		54.5	55.0	43.9	44.6	46.4	44.8	51.1	43.4	44.4	43.6	46.6	44.2
(11) Trout-b	233	69.5	67.4	68.7	67.0	68.2	67.4	68.7	68.2	61.4	66.1		98.7	53.8	49.8	54.5	54.0	50.6	44.7	42.9	44.1	46.7	43.5
(12) Sockeye-b	233	69.1	67.0	68.2	67.0	67.8	67.8	68.2	67.8	62.2	66.5	98.7		52.9	49.8	54.5	54.0	50.8	45.2	41.8	44.4	47.1	43.9
(13) Tetraodon-b	214	60.6	60.6	59.7	61.9	62.3	61.9	61.0	61.5	57.6	58.4	66.5	65.2		69.7	58.0	54.1	42.9	39.3	37.4	40.2	37.4	38.8
(14) Fugu-b	214	58.2	58.2	56.9	63.2	63.6	61.8	61.9	58.8	62.1	62.6	63.1	63.1	80.1		60.5	57.4	43.5	37.9	36.4	38.3	38.9	37.9
(15) Stickleback-b	210	60.4	60.4	59.6	61.8	64.4	61.8	63.3	59.7	60.4	61.7	66.1	66.1	69.3	74.8		67.1	50.0	41.8	42.7	42.4	40.9	40.8
(16) Medaka-b	201	62.2	61.8	62.2	60.9	60.9	61.8	62.4	61.9	65.6	60.4	66.1	66.1	67.1	73.4	78.1		47.2	39.2	39.6	39.5	41.0	37.5
(17) Zebrafish-b	204	64.4	64.9	64.9	68.6	65.3	63.1	62.8	62.8	68.4	67.6	63.1	63.1	59.3	60.3	64.3	68.6		38.5	37.7	38.2	39.0	38.4
(18) Human	258	53.5	57.0	53.9	56.2	57.8	58.5	57.0	56.6	53.9	57.0	57.8	58.1	54.3	50.4	53.1	50.0	50.4		90.7	89.9	66.5	56.6
(19) Mouse	257	52.1	54.5	52.5	54.5	56.8	56.8	56.0	56.8	52.1	56.8	56.8	57.2	53.3	49.0	56.0	52.1	50.6	92.2		88.7	66.4	55.8
(20) Cow	254	55.1	55.9	55.5	53.9	58.7	57.1	59.4	55.1	53.1	57.5	57.1	58.7	54.3	50.0	55.1	52.8	51.2	91.5	90.3		65.3	57.0
(21) Chicken	249	59.4	58.2	59.8	56.6	61.0	58.2	57.4	58.6	54.6	60.2	61.4	61.0	56.6	52.2	53.8	53.4	53.8	76.0	75.9	76.8		60.6
(22) Frog	257	54.9	55.6	55.6	53.7	57.2	53.7	56.8	54.1	56.0	56.8	56.0	57.2	54.5	52.9	53.7	52.1	50.2	75.2	73.5	73.2	73.5	

**Table 2:** Amino acid identities (top right) and similarities (bottom left) of the SOCS2 molecules from fish, birds, amphibians, and mammals. The accession numbers of the protein sequences are given in Figure 3.

	aa	1	2	3	4	5	6	7	8	9	10	11	12	13
(1) Trout-a	201		41.3	24.1	79.1	79.1	74.1	73.6	74.1	51.2	51.2	51.2	47.3	55.8
(2) Trout-b	218	59.2		38.5	40.8	41.7	42.5	41.6	42	40.8	40.5	39.9	38.5	41.3
(3) Trout-bRel	110	36.3	39.9		23.9	24.4	26.2	25.1	23.9	25.8	25.3	24.7	24.2	25.9
(4) gCarp	197	89.6	60.6	35.5		95.4	75.3	73.1	71.6	57.9	57.9	57.9	49.5	54.1
(5) Zebrafish	197	89.6	60.1	35.5	98.5		75.1	72.5	71.1	57.9	56.4	57.9	50.5	53.9
(6) Stickleback	195	82.1	58.7	36.4	86.3	85.3		75.1	75.1	53.5	55.7	53.7	46.5	57.4
(7) Tetraodon	201	81.6	58.7	37.3	85.6	86.6	82.6		88.1	52.4	51.4	52.4	46.1	52.4
(8) Fugu	201	80.6	61	35.3	83.1	83.6	81.6	92		51.2	51.2	50.7	45.6	53.8
(9) Human	198	66.7	56.9	34.8	72.2	71.7	70.7	69.7	68.2		94.4	93.9	61.7	62.9
(10) Cow	198	69.2	57.8	35.4	72.7	71.7	72.2	70.6	68.2	96		92.9	61.9	62.4
(11) Mouse	198	68.2	57.3	34.3	72.7	72.2	70.2	68.7	66.2	95.5	94.4		61.6	61.4
(12) Chicken	207	63.3	56	32.9	64.7	64.3	63.3	62.8	63.8	75.4	76.3	74.9		60.4
(13) Frog	201	74.6	61.5	34.3	72.1	72.1	72.6	70.6	71.6	80.1	79.1	78.6	74.9	

**Table 3:** Amino acid identities (top right) and similarities (bottom left) of the fish SOCS5a and 5b molecules and SOCS5 molecules from birds, amphibians, and mammals. The accession numbers of the protein sequences are given in Figure 3.

	aa	1	2	3	4	5	6	7	8	9	10	11	12	13	14	15	16	17
1. Zebrafish-a1	557		62.8	76.3	76.3	76.6	75.0	42.7	43.2	43.3	43.0	41.3	41.9	64.1	64.0	64.6	63.1	60.2
2. Zebrafish-a2	528	76.1		64.8	66.3	64.4	64.2	44.5	42.9	43.1	42.7	43.3	42.4	63.1	63.1	62.5	62.2	60.1
3. Stickleback-a	561	84.5	75.6		87.4	86.4	84.8	42.3	43.6	42.4	43.9	41.9	43.2	65.4	65.4	65.9	63.9	62.4
4. Medaka-a	561	84.5	76.3	91.1		83.2	82.8	43.3	42.0	42.5	44.4	43.5	42.1	64.4	64.6	66.3	65.1	62.3
5. Tetraodon-a	559	83.5	75.3	90.4	87.9		90.5	42.5	42.4	44.1	43.1	40.7	42.4	63.4	63.6	65.2	63.8	61.5
6. Fugu-a	550	83.8	76.4	89.1	88.1	93.7		43.0	43.0	43.3	43.7	41.4	43.5	61.5	62.0	63.6	63.2	61.4
7. Zebrafish-b	497	57.5	59.7	56.7	55.8	57.4	57.3		63.7	63.5	61.5	61.6	61.2	44.5	44.8	45.6	44.4	44.8
8. Trout-b	544	60.7	60.7	59.0	59.5	58.5	59.1	75.0		74.4	75.0	71.5	72.1	43.9	44.9	45.4	43.9	42.8
9. Stickleback-b	533	60.7	59.5	57.8	58.1	60.5	59.3	76.4	81.6		76.5	73.1	73.3	45.4	45.7	44.3	45.4	44.1
10. Medaka-b	544	60.9	59.0	59.4	59.4	58.1	59.1	74.3	84.9	84.2		74.6	73.7	45.5	47.1	45.8	46.2	45.3
11. Tetraodon-b	542	59.8	59.8	57.8	59.5	56.4	58.4	72.7	80.7	80.1	82.4		88.2	44.5	45.6	46.1	44.1	42.1
12. Fugu-b	537	59.2	59.0	59.2	58.5	57.6	59.6	72.6	81.6	81.4	82.0	90.8		44.2	44.6	44.9	43.6	43.8
13. Human	536	76.1	75.4	75.2	74.7	75.1	75.1	58.8	61.4	63.4	63.1	61.4	62.2		97.8	94.6	91.0	80.6
14. Cow	536	75.8	75.6	75.4	74.9	74.8	75.5	59.3	61.6	62.3	63.2	61.6	62.2	99.1		95.0	90.9	80.1
15. Mouse	536	76.5	75.9	75.6	75.0	75.1	76.2	60.8	61.8	63.2	63.8	61.8	62.6	97.2	97.8		89.4	79.0
16. Chicken	536	74.3	75.6	74.7	74.3	75.3	76.2	58.6	61.2	61.2	63.1	60.9	61.8	95.1	94.8	94.4		79.7
17. Frog	535	74.7	75.5	74.2	74.9	74.1	75.6	59.3	60.1	63.6	61.6	60.3	62.2	90.1	89.6	89.2	88.8	
